# Combination of Broccoli Sprout Extract and Zinc Provides Better Protection against Intermittent Hypoxia-Induced Cardiomyopathy Than Monotherapy in Mice

**DOI:** 10.1155/2019/2985901

**Published:** 2019-12-14

**Authors:** Jiqun Wang, Jian Zhang, Liping Chen, Jun Cai, Zhijie Li, Zhiguo Zhang, Qi Zheng, Yonggang Wang, Shanshan Zhou, Quan Liu, Lu Cai

**Affiliations:** ^1^The Center of Cardiovascular Diseases, The First Hospital of Jilin University, Changchun 130021, China; ^2^Pediatric Research Institute, Department of Pediatrics, University of Louisville, Louisville, KY 40202, USA; ^3^Department of Echocardiography, The First Hospital of Jilin University, Changchun 130021, China; ^4^Department of Pediatrics, The Second Affiliated Hospital and Yuying Children's Hospital of Wenzhou Medical University, Wenzhou 325000, China; ^5^Department of Bioinformatics and Biostatistics, University of Louisville, Louisville, KY 40202, USA; ^6^Departments of Radiation Oncology, Pharmacology & Toxicology, University of Louisville, Louisville, KY 40202, USA

## Abstract

Nuclear factor-E2-related factor 2 (Nrf2) and metallothionein have each been reported to protect against chronic intermittent hypoxia- (IH-) induced cardiomyopathy. Sulforaphane-rich broccoli sprout extract (BSE) and zinc can effectively induce Nrf2 and metallothionein, respectively, to protect against IH-induced cardiomyopathy via antioxidative stress. However, whether the cardiac protective effects of the combination of BSE and zinc can be synergistic or the same has not been evaluated. In this study, we treated 8-week-old C57BL/6J mice with BSE and/or zinc during exposure to IH for 8 weeks. Cardiac dysfunction, as determined by echocardiography, and pathological remodeling and abnormalities, including cardiac fibrosis, inflammation, and oxidative damage, examined by histopathology and western blotting, were clearly observed in IH mice but were not significant in IH mice treated with either BSE, zinc, or zinc/BSE. Furthermore, the effects of the combined treatment with BSE and zinc were always greater than those of single treatments. Nrf2 function and metallothionein expression in the heart increased to a greater extent using the combination of BSE and zinc than using BSE or zinc alone. These findings for the first time indicate that the dual activation of Nrf2 and metallothionein by combined treatment with BSE and zinc may be more effective than monotherapy at preventing the development of IH-induced cardiomyopathy.

## 1. Introduction

Obstructive sleep apnea syndrome is one of the most common breathing disorders in sleep, with a high prevalence of 3–7% and severe consequences [1]. It is characterized by intermittent hypoxia (IH) due to recurrent episodes of partial or complete collapse of the upper airway during sleep [1], leading to blood hypoxemia, hypercapnia, sleep fragmentation, augmented respiratory efforts, and increased sympathetic activity [2]. Obstructive sleep apnea is an independent risk factor for multiple cardiovascular diseases, such as hypertension, coronary artery disease, chronic heart failure, and arrhythmias [3–5]. Long-term IH can cause left ventricular (LV) remodeling and dysfunction and can promote ventricular arrhythmias and sudden cardiac death [6, 7].

Although several underlying mechanisms have been proposed, oxidative stress is currently considered the major mechanism by which IH causes cardiac damage. Oxidative stress represents an imbalance between the production of reactive oxygen species (ROS) and/or reactive nitrogen species (RNS) and the antioxidant capacity of a biological system [8]. Many studies using animal models have confirmed the hypothesis that oxidative stress plays a critical role in the pathogenesis of chronic IH-induced cardiomyopathy [9–11] and causes DNA fragmentation, apoptosis, and autophagy [12]. Therefore, treatments targeting oxidative stress may be suitable for the effective prevention of chronic IH-induced cardiac damage.

Nuclear factor-E2-related factor 2 (Nrf2) is a cap‘n'collar basic-region leucine zipper transcription factor with high sensitivity to oxidative stress [13]. It can upregulate a variety of antioxidant genes via binding to antioxidant response elements (AREs) in the nucleus and protect cells from various injuries, thus influencing the course of disease. Kelch-like ECH-associated protein 1 (Keap1) is a substrate adaptor for cullin-based E3 ubiquitin ligase, which binds Nrf2 and inhibits Nrf2 transcriptional activity via ubiquitination and proteasomal degradation in the cytosol under normal conditions [14]. However, under oxidative stress conditions, Nrf2 dissociates from Keap1 and subsequently translocates into the nucleus, where Nrf2 binds to AREs in the promoter of downstream targets [15, 16]. We have previously demonstrated that Nrf2 can ameliorate IH-induced oxidative stress, thereby preventing cardiac remodeling and dysfunction [17, 18]. Sulforaphane (SFN) is a chemical Nrf2 activator and is stored as its relatively stable precursor glucoraphanin (GRN) in a variety of cruciferous plants, among which broccoli is the most abundant [19]. A group at the Johns Hopkins University found that 3-day-old sprouts of cultivars of certain crucifers contain 10–100 times higher concentrations of GRN than those in the corresponding mature plants [20]. Therefore, increasing research has focused on broccoli sprout extract (BSE), demonstrating that this natural SFN-rich supplement has antioxidative effects by upregulating Nrf2 [21].

Metallothionein (MT) is a cysteine-rich and heavy metal-binding protein with low molecular weight [22]. It is also a potent scavenger of free radicals as a result of its high thiol content and zinc (Zn) which is one of the efficient activators [23, 24]. Accordingly, MT has effective protective effects against IH-induced cardiac damage [17, 25].

BSE and Zn effectively protect against organ or tissue damage caused by oxidative stress via different molecular targets (Nrf2 and MT, respectively) [26–29]. We have previously reported that the combination of SFN and Zn achieves better prevention of diabetic cardiomyopathy in type 1 diabetic OVE26 mice [30]. However, mechanisms by which IH and diabetes induce organ damages are significantly different. Our hypothesis is that simultaneous induction of both Nrf2 and MT may provide a better or synergistic protection against IH-induced cardiomyopathy than induction of Nrf2 or MT alone. In addition, considering the advantage of vegetable extract BSE that has more potential to be clinically applied than SFN (a pure compound), we decided to combine the treatment of BSE as the Nrf2 inducer with Zn as the MT inducer to evaluate this hypothesis. Therefore, we used a chronic IH model to compare the effects of Zn, BSE, and their combination on IH-induced cardiac function and pathogenic mechanisms.

## 2. Materials and Methods

### 2.1. Animals and IH Exposure

C57BL/6J mice were housed at the University of Louisville Research Resources Center at 22°C with a 12 h light/dark cycle with free access to standard rodent chow and water. All animal procedures followed the NIH *Guide for the Care and Use of Laboratory Animals* and were approved by the University of Louisville Institutional Animal Care and Use Committee (IACUC number: 15008). A previously described murine model of IH exposure during sleep was used [31]. Briefly, the IH paradigm consisted alternating cycles of 20.9% O_2_/8% O_2_ FiO_2_ (30 episodes per hour) lasting 20 s at the nadir of FiO_2_ during the 12 h light phase. In this study, 8-week-old male mice were divided into two groups for 8 weeks of exposure, an IH group and a room air control group.

### 2.2. *In Vivo* Administration of BSE and Zn

Mice were administered with BSE (equivalent to SFN 2 mg/kg; CS Health, LLC, Louisville, KY, USA) and/or Zn sulfate heptahydrate (5 mg/kg; Sigma-Aldrich, St. Louis, MO, USA) by gavage from 8 weeks of age at a frequency of once every other day for 8 weeks. The doses of BSE and Zn were based on previous studies [21, 24]. BSE was crushed and then suspended in carboxymethyl cellulose, and Zn sulfate was dissolved in 1% dimethyl sulfoxide. Vehicle control mice were administered an equivalent volume of PBS containing 1% dimethyl sulfoxide. According to the conversion of animal doses to human equivalent doses based on body surface area guided by the FDA [32], the doses used in this study are safe to convert to human doses.

### 2.3. Echocardiography

Transthoracic echocardiography was performed using a high-resolution imaging system (Vevo 770; Visual Sonics, Toronto, ON, Canada) to assess cardiac function, as described previously [33]. Briefly, mice were anesthetized with isoflurane and placed in the supine position on a heating pad (100% O_2_; 0.5 L/min). Two-dimensional and M-mode echocardiography was used to assess wall motion, chamber dimensions, and cardiac function.

### 2.4. Sirius Red Staining

Heart paraffin sections and Sirius Red staining were processed as previously described [30, 34].

Tissue containing collagen was stained red, and myocardial tissue was stained green. ImageJ (National Institutes of Health, Bethesda, MD, USA) was used to analyze red areas in order to estimate the collagen content.

### 2.5. Western Blotting

Western blotting was performed as described previously [30, 35], and MT expression was detected using a modified western blotting protocol [24]. Primary antibodies against the following were used: 3-nitrotyrosine (3-NT; 1 : 1,000; Millipore, Billerica, MA, USA); Nrf2 (1 : 1,000; Abcam, Cambridge, MA, USA); *α*-smooth muscle actin (*α*-SMA; 1 : 1,000; Abcam); connective tissue growth factor (CTGF; 1 : 1,000; Santa Cruz Biotechnology, Santa Cruz, CA, USA); fibronectin (1 : 1,000; Abcam); tumor necrosis factor *α* (TNF-*α*; 1 : 1,000; Abcam); NACHT, LRR, and PYD domain-containing protein 3 (NLRP3; 1 : 1,000; Abcam); NAD(P)H:quinone oxidoreductase 1 (NQO1; 1 : 1,000; Santa Cruz Biotechnology); superoxide dismutase-2 (SOD-2; 1 : 5,000; Santa Cruz Biotechnology); catalase (CAT; 1 : 5,000; Santa Cruz Biotechnology); *β*-actin (1 : 8,000; Santa Cruz Biotechnology); and MT (1 : 1,000; DakoCytomation, Carpinteria, CA, USA).

The protein content was determined by measuring the gray values of bands using Image Lab (Bio-Rad).

### 2.6. Statistical Analysis

Data are expressed as the means and standard deviation (SD) for each outcome variable. The *t*-test was performed to identify statistically significant differences between the means of two groups. One-way analysis of variance (ANOVA) followed by the Tukey *post hoc* test was used to identify differences among the means of multiple groups. The normality assumption required by the *t-*test and the one-way ANOVA was assessed by the Shapiro–Wilk test. When this assumption was violated, the commonly used log transformation was then applied. In addition, the condition of equal variances for the one-way ANOVA was validated by Levene's test. The sample sizes in our study were justified by calculating effect sizes. Given the large effect sizes in the comparisons, the *t-*test or one-way ANOVA has at least 80% power to detect a meaningful difference at a significance level of 0.05. The statistical analyses and generation of plots were performed using GraphPad Prism 7 (GraphPad Software Inc., San Diego, CA, USA). Results were considered statistically significant when *P* < 0.05.

## 3. Results

### 3.1. Characterization of Cardiomyopathy in IH Mice

**Figure 1:**
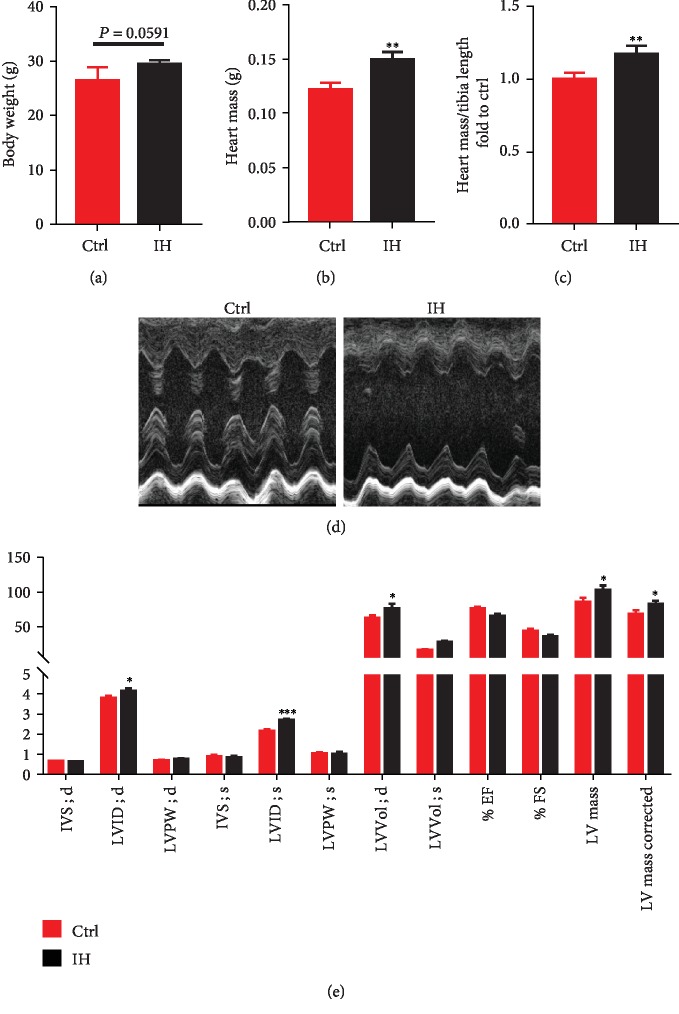
Cardiac structure and function in intermittent hypoxia (IH) mice. C57BL/6J mice were used to establish a model of IH. (a) Body weight immediately prior to sacrifice. (b, c) Heart mass and heart mass/tibia length ratio. (d, e) Cardiac function, as assessed by echocardiography. Data are presented as the means ± SD (*n* = 4). ^∗^*P* < 0.05*vs*. control mice; ^∗∗^*P* < 0.01*vs*. control mice; ^∗∗∗^*P* < 0.001*vs*. control mice.

There was no significant difference in body weight between control and IH-exposed mice (Figure 1(a)). Compared with those of the control group, the heart mass (Figure 1(b)) and heart mass/tibia length ratio (Figure 1(c)) in the IH group were significantly higher. Echocardiography showed that, compared with those in the control group, the LV internal diastolic diameter (LVID; d), LV internal systolic diameter (LVID; s), LV diastolic volume (LV Vol; d), LV systolic volume (LV Vol; s), and LV mess were significantly higher in the IH group, while ejection fraction (EF) and fractional shortening (FS) were significantly lower in the IH group than those in the control group (Figures 1(d) and 1(e)). This indicates that IH causes cardiac hypertrophy and cardiac dysfunction.

**Figure 2:**
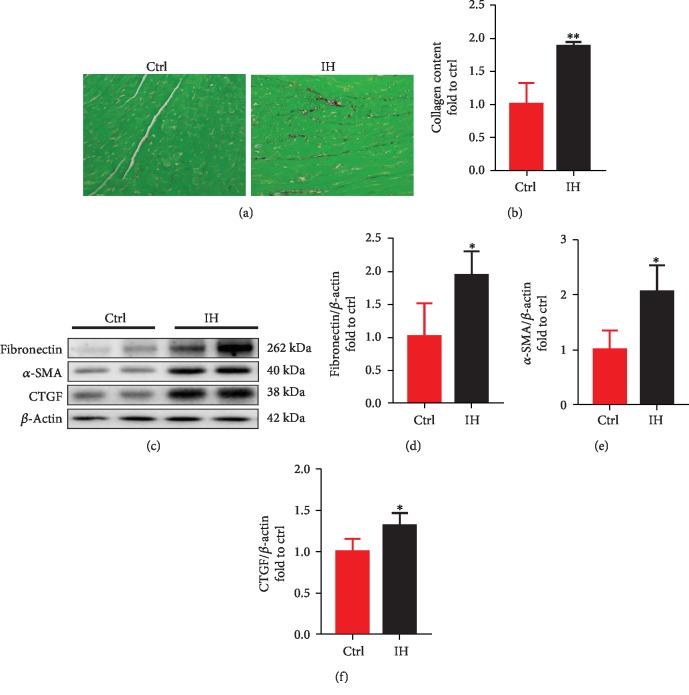
Cardiac fibrosis is significantly more severe in IH mice than in controls. (a, b) Cardiac collagen deposition was assessed by Sirius Red staining. (c–f) Cardiac fibrosis was also assessed by western blotting analyses of fibronectin, *α*-smooth muscle actin (*α*-SMA), and connective tissue growth factor (CTGF). Data are presented as the means ± SD (*n* = 4). ^∗^*P* < 0.05*vs*. control mice; ^∗∗^*P* < 0.01*vs*. control mice.

Using Sirius Red staining, we found that collagen deposition was significantly higher in the IH group than in the control group (Figures 2(a) and 2(b)). As determined by western blotting, the expression levels of fibronectin, *α*-SMA, and CTGF in the IH group mice were also significantly higher than those in the control group (Figures 2(c)–2(f)). These findings indicate that cardiac fibrosis in IH mice is consistent with the presence of cardiac hypertrophy and dysfunction (i.e., cardiomyopathy) observed in our previous studies [17].

### 3.2. Therapeutic Effects of BSE and/or Zn in IH Mice

#### 3.2.1. General Effects, Cardiac Structure, and Function

**Figure 3:**
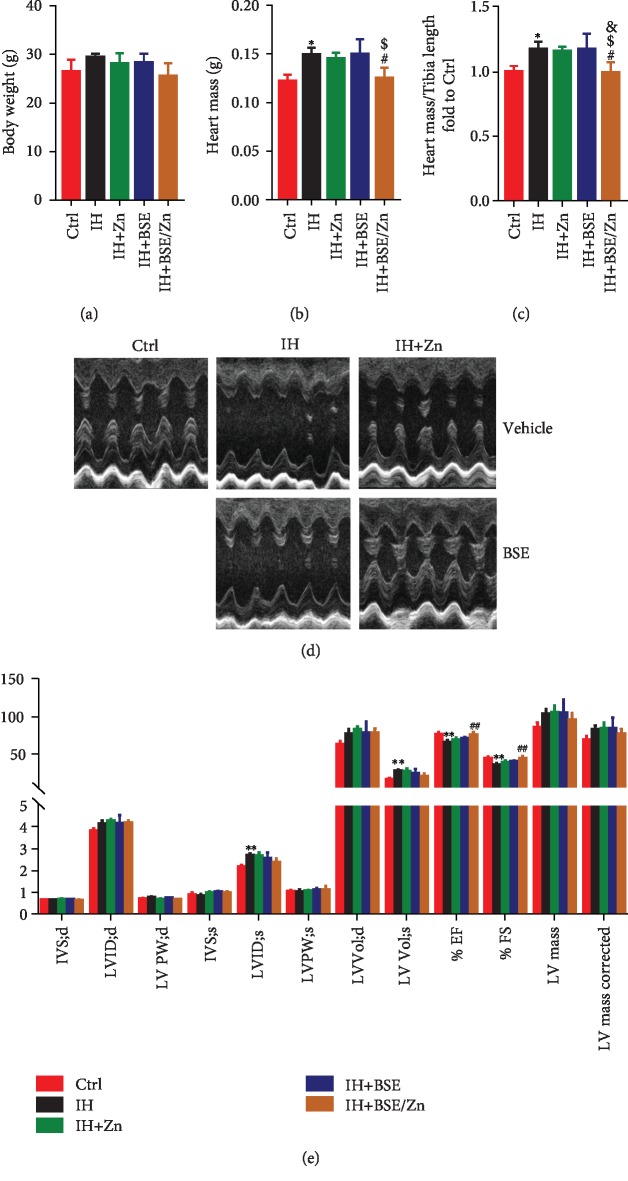
Broccoli sprout extract (BSE) and/or zinc (Zn) treatment ameliorates IH-associated defects in cardiac structure and function. Mice were administered with BSE and/or Zn by gavage at doses of 2 mg/kg and 5 mg/kg, respectively, every other day for 8 weeks, from 8 weeks of age. Vehicle control mice were administered an equivalent volume of phosphate-buffered saline (PBS) containing 1% dimethyl sulfoxide. (a) Body weight. (b, c) Heart mass and heart mass/tibia length ratio. (d, e) Cardiac function, as assessed by echocardiography. Data are presented as the means ± SD (*n* = 4). ^∗^*P* < 0.05*vs*. control group mice; ^∗∗^*P* < 0.01*vs*. control group mice; ^#^*P* < 0.05*vs*. IH group mice; ^##^*P* < 0.01*vs*. IH group mice; ^&^*P* < 0.05*vs*. IH+Zn group mice; ^$^*P* < 0.05*vs*. IH+BSE group mice.

There were no significant effects of either Zn, BSE, or Zn/BSE on body weight in IH-exposed mice (Figure 3(a)). However, the heart mass was significantly lower in the IH-BSE/Zn group than in the IH and IH-BSE groups (Figure 3(b)), while the heart mass/tibia length ratio was significantly lower in the IH-BSE/Zn group than in the IH and monotherapy groups (Figure 3(c)). With respect to cardiac structure and function, compared with the IH group, there was no significant difference in the monotherapy groups, but the EF and FS were significantly higher in the combined treatment group (Figures 3(d) and 3(e)). Thus, the echocardiography data indicate that combined treatment with Zn/BSE ameliorates IH-induced defects in cardiac structure and function.

#### 3.2.2. Treatment with BSE and Zn Ameliorates Myocardial Fibrosis

**Figure 4:**
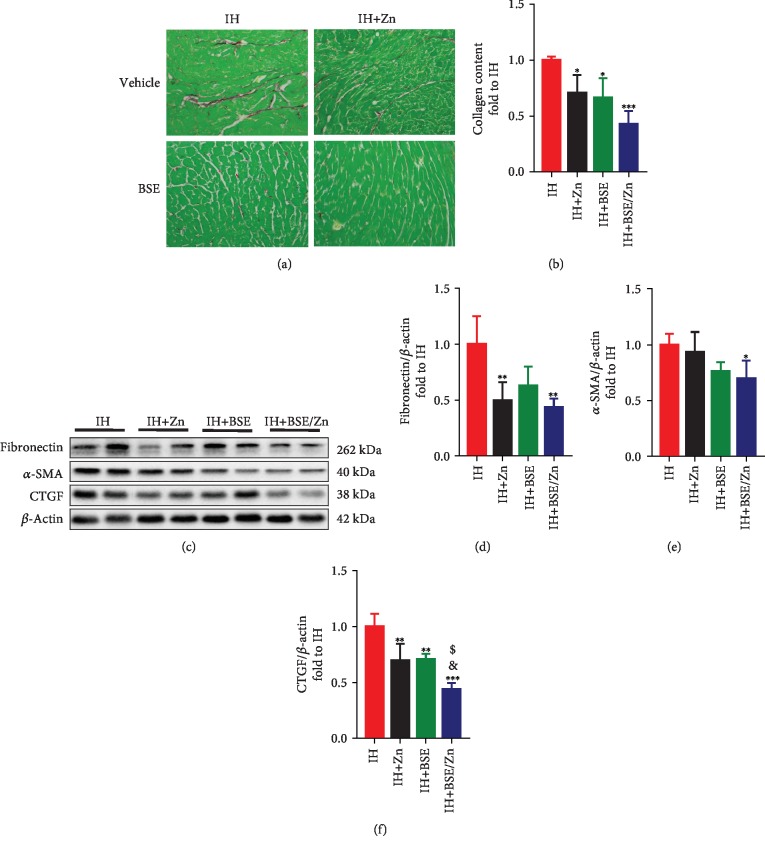
BSE and/or Zn treatment ameliorates cardiac fibrosis. (a, b) Cardiac collagen deposition was assessed by Sirius Red staining. (c–f) Cardiac fibrosis was assessed by western blotting analyses of fibronectin, *α*-SMA, and CTGF. Data are presented as the means ± SD (*n* = 4). ^∗^*P* < 0.05*vs*. IH group mice; ^∗∗^*P* < 0.01*vs*. IH group mice; ^∗∗∗^*P* < 0.001*vs*. IH group mice; ^&^*P* < 0.05*vs*. IH+Zn group mice; ^$^*P* < 0.05*vs*. IH+BSE group mice.

Cardiac fibrosis is an important indicator of cardiac dysfunction and structural changes. Based on Sirius Red staining, reduced myocardial collagen deposition was observed in IH mice in all treatment groups, with the greatest reduction in the combined treatment group (Figures 4(a) and 4(b)). Western blotting showed that fibronectin protein levels were significantly lower in the Zn and combined groups than in other groups (Figures 4(c) and 4(d)) but tended to be lower in the BSE group than in the IH alone group. The expression of *α*-SMA tended to be low in the monotherapy groups and was significantly lower in the BSE/Zn-treated group than in the IH group (Figures 4(c) and 4(e)). CTGF levels were significantly lower in all treatment groups than in the IH group, but the combined treatment showed the greatest reduction in the IH-increased expression of CTGF (Figures 4(c) and 4(f)). These data demonstrate that treatment with BSE and/or Zn can ameliorate myocardial fibrosis associated with IH, to a certain extent, and that combination therapy has the best antifibrotic effect among the treatments.

### 3.3. Mechanism Underlying the Preventive Effects of BSE and Zn

#### 3.3.1. Anti-Inflammatory and Antioxidative Stress Effects

**Figure 5:**
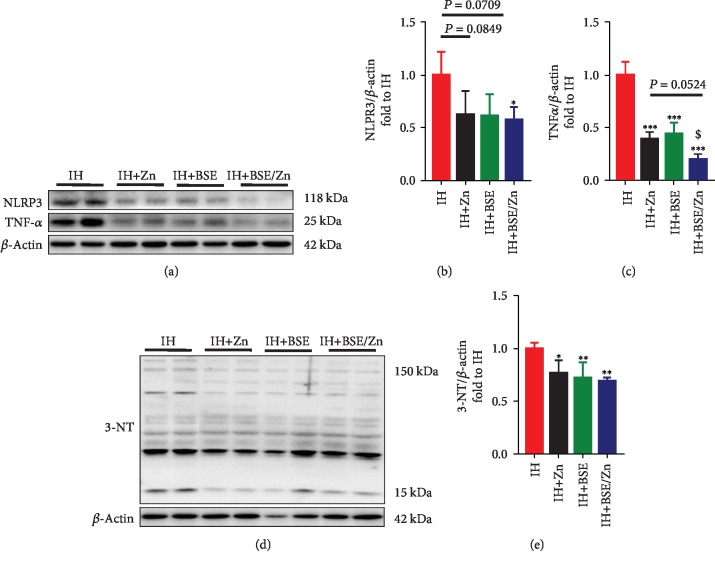
BSE and/or Zn treatment protects against inflammation and oxidative stress induced by IH. (a–c) Cardiac inflammation was assessed by western blotting analyses of NACHT, LRR, and PYD domain-containing protein 3 (NLRP3) and tumor necrosis factor *α* (TNF-*α*). Data are presented as the means ± SD (*n* = 4). (d, e) Cardiac oxidative damage was assessed using western blotting analyses of 3-nitrotyrosine (3-NT). Bands of 3-NT were analyzed from 150 kDa to 15 kDa. ^∗^*P* < 0.05 vs. IH group mice; ^∗∗^*P* < 0.01*vs*. IH group mice; ^∗∗∗^*P* < 0.001*vs*. IH group mice; ^$^*P* < 0.05*vs*. IH+BSE group mice.

Cardiac fibrosis is predominantly stimulated by chronic inflammation; therefore, we examined whether the combined treatment prevents cardiac inflammation in IH-treated mice. As determined by western blotting, the NLRP-3 level was slightly lower after monotreatment by Zn or BSE (*P* > 0.05) and significantly lower after combined treatment (*P* < 0.05) than that in the IH group (Figures 5(a) and 5(b)). However, Zn or BSE monotherapy and combined treatment all significantly reduced TNF-*α* expression, with the combined treatment showing a further significant reduction compared with Zn monotherapy (Figures 5(a) and 5(c)). These results show that BSE or Zn can significantly ameliorate myocardial inflammation induced by IH, but the combination provides a better anti-inflammatory effect.

Inflammation is a response to oxidative stress, and both oxidative stress and inflammation can be exacerbated by each other [11, 36, 37]. Accordingly, we used 3-NT as an indicator of the severity of oxidative stress. 3-NT protein levels were significantly reduced in IH mice for all treatment groups, and the reduction was greater in the combination treatment group (Figures 5(d) and 5(e)).

#### 3.3.2. Activation of Nrf2 and Its Downstream Targets

**Figure 6:**
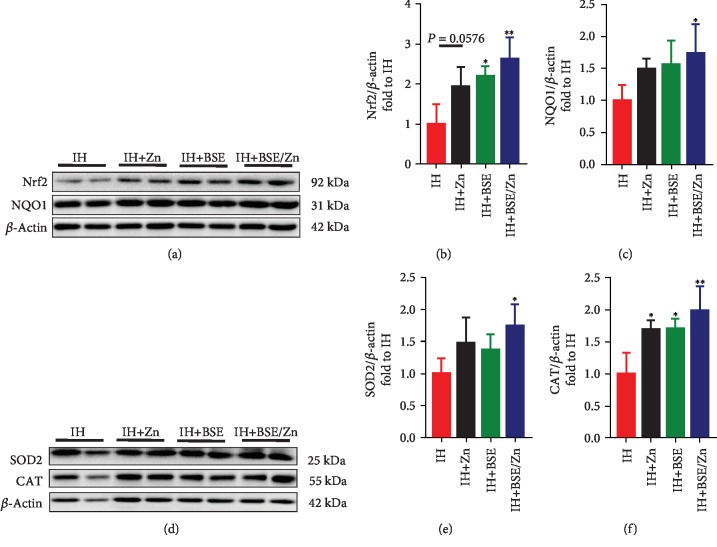
BSE and/or Zn treatment increases the protein expression levels of nuclear factor E2-related factor 2 (Nrf2) and its downstream targets. The expression levels of Nrf2, NAD(P)H:quinone oxidoreductase 1 (NQO1), superoxide dismutase-2 (SOD-2), and catalase (CAT) were measured by western blotting. Data are presented as the means ± SD (*n* = 4). ^∗^*P* < 0.05*vs*. IH group mice; ^∗∗^*P* < 0.01*vs*. IH group mice.

Nrf2 via its downstream antioxidants has important antioxidant effects. We found that total Nrf2 expression is significantly higher in the BSE and combined treatment groups (*P* < 0.05) but only slightly higher in the Zn-treated group (*P* > 0.05) than in the IH group (Figures 6(a) and 6(b)).

Numerous studies have shown that Nrf2 downstream targets, such as NQO-1, CAT, and SOD, have significant antioxidant effects on various cardiovascular diseases [38–41]. Therefore, we also measured the expression of NQO-1, CAT, and SOD at the protein level (Figures 6(a) and 6(c)–6(f)). NQO-1 and SOD-2 levels tended to be higher in monotherapy groups and were significantly higher in the combined treatment group than in the IH group (Figures 6(a) and 6(c)–6(e)). In all treatment groups, CAT expression was significantly higher than that in the IH group but the increase tended to become higher in the combined treatment group than in the monotreatment groups (Figures 6(d) and 6(f)). These results suggest that the combination is more effective than monotherapies to activate Nrf2-mediated antioxidant function.

#### 3.3.3. Upregulation of MT Expression

**Figure 7:**
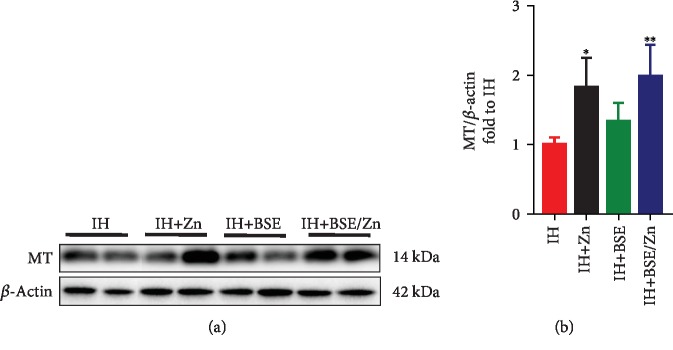
BSE and/or Zn treatment increases the protein expression of metallothionein (MT). Protein expression of MT measured by western blotting. Data are presented as the means ± SD (*n* = 4). ^∗^*P* < 0.05*vs*. IH group mice; ^∗∗^*P* < 0.01*vs*. IH group mice.

The expression of MT was also measured in each group at the protein level by a western blotting assay (Figure 7). In the Zn-treated and combination treatment groups, MT protein expression was significantly higher than that in the IH group and there was only a slight increase in the IH-BSE group compared to the IH group. This suggests that MT protein expression predominantly responds to Zn treatment compared to BSE treatment.

## 4. Discussion

In previous studies, Nrf2 has typically been induced using SFN or SFN-rich BSE [21, 33], and MT has predominantly been induced using Zn [42]. However, studies of the combined use of Nrf2 and MT activators for the prevention of IH-induced cardiomyopathy are lacking. In this study, we evaluated, for the first time, the inducer of Nrf2 or MT alone and in combination to show that (1) BSE or Zn each provide some degree of protection from IH-induced cardiomyopathy and (2) compared with activators of Nrf2 or MT alone, the combination provides more significant protection from IH-induced cardiomyopathy. Our results provide further support for the roles of Nrf2, its downstream targets, and MT in antioxidative stress to prevent IH-induced cardiotoxicity.

Broccoli sprouts are rich sources of glucosinolates and their hydrolysis products. The concentration of GRN, the main glucosinolate in broccoli sprouts, is 15 times greater than that in mature plants, and the content of SFN, a degradation product of GRN, is 10 times higher than that of mature plants [20, 43]. Some studies have shown that broccoli sprouts could reduce cardiac damage, including oxidative stress caused by ischemia-reperfusion and type 2 diabetes [21, 44]. Therefore, several kinds of broccoli extracts, including BSE, have been utilized in clinical trials to explore the potential clinical applications [45–47].

Combination therapies are commonly used in clinical settings. SFN and 5-fluorouracil synergistically reduce cell growth in a triple-negative breast cancer line by inducing autophagic cell death and premature senescence [48]. Epidermal squamous cell carcinoma and malignant mesothelioma can be treated using a combination of SFN and cisplatin, inhibiting tumor cell proliferation and accelerating tumor cell autophagy [49, 50]. Furthermore, the combination of SFN and myricetin can induce fat cell apoptosis via Akt-mediated mitochondrial apoptosis [51], suggesting a novel strategy for the treatment of obesity. Finally, in inflammatory bowel disease, the combination of SFN and selenium synergistically upregulates TrxR-1, which plays an important role in maintaining intracellular redox homeostasis and contributes to SFN-induced protection against free radical-mediated oxidative damage in normal colon cells [52].

We have found that mice with an *mt* gene deletion are highly susceptible and mice with cardiac overexpression of *mt* are resistant to IH-induced cardiomyopathy [17], suggesting the important role of MT in the prevention of IH-induced cardiotoxicity. Therefore, the combination of SFN with Zn may improve the prevention of IH-induced cardiotoxicity. Although the combined use of SFN with Zn to protect against IH-induced pathogenic damage (in particular, cardiotoxicity) has not been evaluated previously, the combination of Zn and other compounds is used to treat several conditions. For example, a combination of Zn and transretinoic acid effectively inhibits the growth of *Listeria monocytogenes* [53]. The combination of Zn and glibenclamide limits cardiovascular complications in diabetic rats [54]. Combination therapies not only permit beneficial drug interactions but can also reduce the adverse effects of drugs, while achieving better therapeutic results.

We did not evaluate the synergistic or additive effects of Zn with SFN on IH-induced cardiomyopathy; instead, we focused on Zn with BSE for the following reasons. (1) Despite several clinical trials of the use of SFN to induce Nrf2 in several diseases [55–57], SFN is not yet applied in clinical settings. (2) BSE is a natural product derived from broccoli sprouts and is commercially available as a health supplement. (3) We have experimentally confirmed the similarity of the effects of SFN and BSE on the development of cardiomyopathy in type 2 diabetic mice [21]. Using the combination of Zn and BSE, we observed synergistic protection from IH-induced cardiomyopathy compared to the effects of monotreatment with either Zn or BSE. Mechanistically, we found that the cardiac protection by Zn and BSE from IH-induced cardiomyopathy is associated with significant increases in MT- and Nrf2-mediated antioxidants, including NQO-1, SOD-2, and CAT.

Our study had a few limitations. For example, the ability of the human gut microflora to hydrolyze GRN and release SFN for absorption in the large intestine varies substantially among individuals [46], but we did not evaluate the kinetics and biodistributions of SFN. We did not compare the control and its treatment groups with IH and its treatment groups owing to the complexity of comparisons among ten groups of mice. We did not further explore crosstalk between Nrf2 and MT to determine whether they synergistically or additively mediate cardiac protection during IH-induced cardiotoxicity. These limitations and the underlying mechanisms will be addressed in future studies.

Moreover, oxidative stress plays an important role not only in IH but also in other diseases such as ischemia-reperfusion injury [58]. Oxidative stress is often associated with elevated levels of ROS or RNS at the cellular and subcellular levels [59]. Ischemia increases ROS at the cellular level, and cardiomyocytes are subjected to further oxidative stress upon reperfusion [60]. This process may lead to arrhythmia, heart failure, and sudden cardiac death [61, 62]. Many studies have shown that some natural plant extracts have protective effects against cardiac ischemia-reperfusion injury. For example, *Ginkgo biloba* extract can improve cardiac function after global ischemia in the isolated working rat heart by reducing the formation of oxygen free radicals [63], and sour cherry seed extract can exert cardioprotective effects by increasing heme oxygenase-1 levels [64]. Zn has also been shown to attenuate lethal reperfusion-induced injury in a manner that is reliant on ErbB2/PI3K/Akt activity [65]. Furthermore, some studies have confirmed that natural plant extracts combined with Zn treatment achieve better results [66, 67].

In summary, our study is the first investigation of the combination of BSE and Zn, Nrf2, and MT inducers, to protect against IH-induced cardiomyopathy. By effectively activating Nrf2, its downstream targets, and MT, this combination can ameliorate the defects associated with IH-induced cardiomyopathy more effectively than monotherapies. These findings suggest new avenues for the future clinical treatment of IH-induced cardiac damage.

## Data Availability

The datasets generated during and/or analyzed during the current study are available from the corresponding authors on reasonable request.
